# Fibrinogen and hemoglobin predict near future cardiovascular events in asymptomatic individuals

**DOI:** 10.1038/s41598-021-84046-7

**Published:** 2021-02-25

**Authors:** Moritz Lassé, Anna P. Pilbrow, Torsten Kleffmann, Elin Andersson Överström, Anne von Zychlinski, Christopher M. A. Frampton, Katrina K. Poppe, Richard W. Troughton, Lynley K. Lewis, Timothy C. R. Prickett, Christopher J. Pemberton, Arthur M. Richards, Vicky A. Cameron

**Affiliations:** 1 Department of Medicine, Christchurch Heart Institute, University of Otago, Christchurch, New Zealand; 2 Department of Biochemistry, University of Otago, Dunedin, New Zealand; 3 Southern Community Laboratories Ltd, Dunedin, New Zealand; 4 School of Population Health, Faculty of Medical and Health Sciences, University of Auckland, Auckland, New Zealand; 5 Cardiovascular Research Institute, National University of Singapore, Singapore, Singapore

**Keywords:** Biochemistry, Biomarkers, Cardiology, Risk factors, Proteomic analysis, ELISA

## Abstract

To identify circulating proteins predictive of acute cardiovascular disease events in the general population, we performed a proteomic screen in plasma from asymptomatic individuals. A “Discovery cohort” of 25 individuals who subsequently incurred a cardiovascular event within 3 years (median age = 70 years, 80% male) was matched to 25 controls remaining event-free for > 5 years (median age = 72 years, 80% male). Plasma proteins were assessed by data independent acquisition mass spectrometry (DIA-MS). Associations with cardiovascular events were tested using Cox regression, adjusted for the New Zealand Cardiovascular Risk Score. Concentrations of leading protein candidates were subsequently measured with ELISAs in a larger (n = 151) independent subset. In the Discovery cohort, 76 plasma proteins were robustly quantified by DIA-MS, with 8 independently associated with cardiovascular events. These included (HR = hazard ratio [95% confidence interval] above vs below median): fibrinogen alpha chain (HR = 1.84 [1.19–2.84]); alpha-2-HS-glycoprotein (also called fetuin A) (HR = 1.86 [1.19–2.93]); clusterin isoform 2 (HR = 1.59 [1.06–2.38]); fibrinogen beta chain (HR = 1.55 [1.04–2.30]); hemoglobin subunit beta (HR = 1.49 [1.04–2.15]); complement component C9 (HR = 1.62 [1.01–2.59]), fibronectin isoform 3 (HR = 0.60 [0.37–0.99]); and lipopolysaccharide-binding protein (HR = 1.58 [1.00–2.49]). The proteins for which DIA-MS and ELISA data were correlated, fibrinogen and hemoglobin, were analyzed in an Extended cohort, with broader inclusion criteria and longer time to events, in which these two proteins were not associated with incident cardiovascular events. We have identified eight candidate proteins that may independently predict cardiovascular events occurring within three years in asymptomatic, low-to-moderate risk individuals, although these appear not to predict events beyond three years.

## Introduction

Cardiovascular disease (CVD) is a leading cause of death and disability worldwide^1^. In 2015 the prevalence of CVD and number of CVD-related deaths was estimated at 423 million and 18 million respectively, with ischemic heart disease (IHD) and stroke contributing the most to loss of age-standardized disability-adjusted life years^1^. Determining an individual’s risk for developing CVD is commonly based on a set of key variables, including age, gender, ethnicity, blood pressure, diabetes mellitus, smoking and lipid status. These established risk factors for CVD have been incorporated into risk prediction models such as the Framingham risk score (FRS), which is used to predict CVD incidence within 10 years^2,3^. Subsequent strategies to improve CVD risk prediction, such as the updated 5-year New Zealand (NZ) primary prevention equations, PREDICT-1°, have incorporated additional variables including the NZ Deprivation score (an area-based measure of socioeconomic deprivation), atrial fibrillation confirmed by electrocardiograph (ECG) and use of blood-pressure-lowering, lipid-lowering, and antithrombotic drugs in the 6 months before the index assessment^4^. The QRISK3 risk prediction model implemented in the UK takes into account 22 risk variables to estimate 10-year CVD risk, with the additional factors being chronic kidney disease, variability of systolic blood pressure, migraine, treatment with corticosteroids, systemic lupus erythematosus, atypical antipsychotic medication, severe mental illness, HIV or AIDS, and erectile dysfunction^5^.

While risk prediction models such as these work well on the population level, they suffer from poor discrimination at the level of the individual. It remains difficult to predict which individuals within any broad risk stratum will subsequently experience a CVD event^6^. CVD events still occur frequently in people predicted to be at low-to-moderate risk^6^. Addition of endogenous biomarkers to CVD risk scores may refine risk stratification for the individual. The only circulating biomarkers routinely incorporated in current CVD risk prediction models are lipids i.e. total cholesterol (TC), high-density lipoprotein (HDL) or their ratio (TC/HDL) and calculated low-density lipoprotein (LDL). Additional biomarker molecules used to diagnose comorbidities in conjunction with CVD risk estimation include glycated hemoglobin (HbA1C) for diabetic status and creatinine for renal status^7,8^. Plasma concentrations of B-type natriuretic peptide (BNP), its amino-terminal pro-hormone congener (NT-proBNP) and the cardiac troponins T and I (TNT, TNI) are key biomarkers in the clinical diagnosis of heart failure (HF) and myocardial infarction (MI), respectively^9–12^. Both markers have also been shown to provide powerful information about CVD events in asymptomatic community populations, with modest elevations in these markers being independently predictive of atherosclerotic CVD, HF, fatal coronary events and total mortality^13–15^.

However, despite some improvement in risk prediction algorithms over the past decades, traditional risk factor profiling fails to identify many individuals at impending risk of an acute CVD event, highlighting the need for new strategies for risk prediction in the general population. We sought to identify novel circulating protein markers associated with incident CVD events in asymptomatic individuals by performing an unbiased proteomics screen using data independent acquisition mass spectrometry (DIA-MS), followed up in an independent cohort using enzyme-linked immunosorbent assays (ELISA).

## Methods

### Study participant recruitment and sample collection

Plasma samples for this case–-control study (n = 50 for the Discovery cohort and n = 151 for the Extended cohort) were selected from the Canterbury Healthy Volunteers cohort (HVols, n = 3,358). HVols participants were randomly selected from the Canterbury (New Zealand) electoral rolls. Participants were screened for prior hospital admissions and cardiac diagnoses. CVD risk factors, anthropometric measures, personal health information and family history of cardiovascular events were recorded for each participant. CVD risk was estimated using both FRS and PREDICT-1° equations^3,4^. Plasma samples were biobanked at − 80 °C and subsequently assayed for high-sensitivity Troponin I (hsTnI) and NT-proBNP. Clinical events of participants have been continuously documented and updated 6-monthly (median follow-up 9.2 years). The study conformed to the Declaration of Helsinki and was approved by the New Zealand Health and Disability Ethics Committee (Reference CTY/01/05/062). All participants gave written, informed consent.

### Patient selection for DIA-MS discovery study

**Figure 1:**
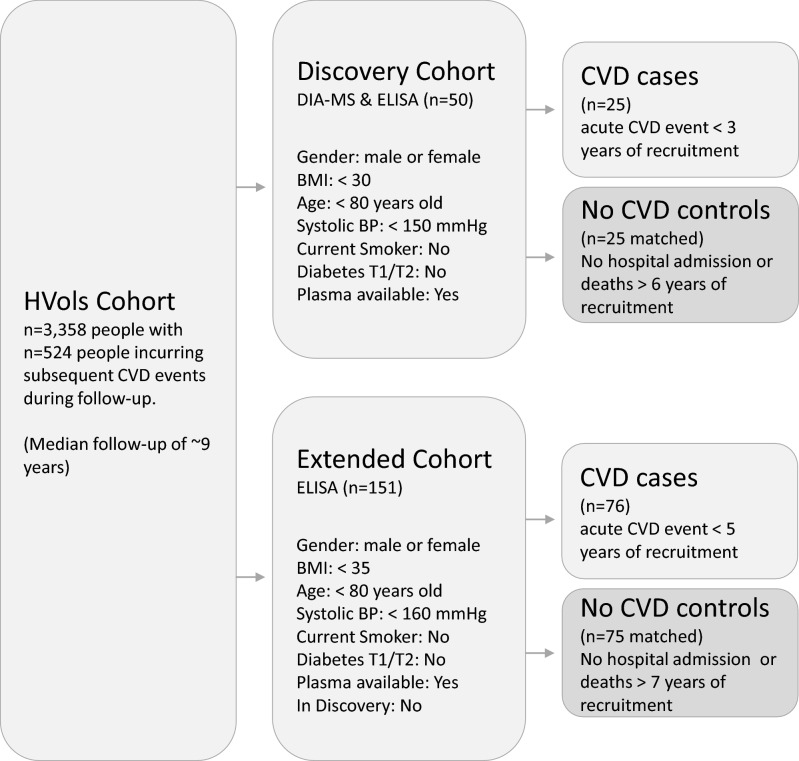
Sample Selection Criteria. The Canterbury Healthy Volunteers cohort (HVols) is a community-based cohort of 3358 individuals of middle to older age with no previously diagnosed cardiovascular disease (CVD). The Discovery cohort included 25 participants who subsequently incurred an acute CVD event within 3 years and 25 who remained event-free for at least 5 years. The Extended cohort was an independent sample of 76 HVols who incurred an acute CVD event within 5 years and 75 controls who remained event-free for at least 7 years. Figure generated using Microsoft PowerPoint 2019.

For the initial Discovery arm of the study using data independent acquisition mass spectrometry (DIA-MS), a subset of HVols aged < 80 years, BMI < 30 kg/m^2^, systolic BP < 150 mmHg, without diabetes and non-smokers, who subsequently experienced an acute CVD event within 3 years were identified (n = 25 cases, Fig. 1). Incident ischemic CVD events were defined as non ST-elevation myocardial infarction, ST-elevation myocardial infarction, ischemic stroke, transient ischemic attack, unstable angina, other angina or death due to coronary heart disease. Matched controls (n = 25) meeting the same criteria but without incurring any CVD events for at least 5 years from recruitment were selected using the R package MatchIt^16^ with “nearest” matching for age, BMI, and systolic blood pressure, and exact matching for gender.

### Patient selection for ELISA extended cohort study

For the Extended arm of the study, a separate subset of healthy volunteers aged < 80 years, BMI < 35 kg/m^2^, systolic BP < 160 mmHg, without diabetes and non-smokers, who experienced an acute CVD event within 5 years were selected (n = 76 cases, Fig. 1). Criteria for the Extended arm had to be relaxed compared with the Discovery Cohort, particularly the time to first event, due to limited sample numbers. Matched controls (n = 75) were selected as described above.

### DIA-MS

To mitigate batch effects a randomized blocked experiment design was used with equal numbers of cases and controls processed at the same time. Plasma was thawed on ice and centrifuged to remove particulate matter. The detailed sample preparation and data analysis methods are outlined in supplementary information. In brief, plasma (2 µL) was denatured (Supplementary Table 1) and hydrolyzed with trypsin. Tryptic peptides were C18 purified and then spiked with retention time calibration peptides to correct for relative retention time differences between runs (Escher et al. 2012). DIA-MS of the 50 individual, trypsinized plasma samples was undertaken using an AB Sciex 5600 + TripleTOF mass spectrometer coupled to an ekspert nanoLC 415 system (eksigent, AB Sciex, Dublin, CA, USA). DIA-MS data were compared to a spectral library generated from pooled samples. Data analysis was carried out with AB Sciex software (SWATH Acquisition MicroApp (version 2.0.0.1) in PeakView (version 2.2) Software) and R (version R-3.6.1) and RStudio (version 1.2.5001)^17,18^.

**Table 1:** Patient characteristics in the Discovery (n = 50) and Extended (n = 151) cohorts.

Variable	Discovery cohort			Extended cohort			Discovery versus Extended
	Controls	Cases	*p* value	controls	cases	*p* value	*p* value
n	25	25		75	76		
Age , years (mean (SD))	67.6 (9.9)	68.3 (10.8)	0.830	69.1 (6.2)	69.3 (7.0)	0.893	0.314
Gender = Male (%)	20 (80)	20 (80)	1	59 (79)	60 (79)	1	1
Ethnicity (%)			1			1	0.685
European	24 (96)	25 (100)		74 (99)	74 (97)		
Māori	0 (0)	0 (0)		1 (1)	0 (0)		
Other	1 (4)	0 (0)		0 (0)	2 (3)		
Smoker = yes (%)	0 (0)	0 (0)	NA	0 (0)	0 (0)	NA	1
BMI [kg/m^2^] (mean (SD))	25.4 (2.3)	24.8 (3.1)	0.412	26.3 (3.4)	26.5 (3.4)	0.847	0.014
SBP [mmHg] (mean (SD))	133.0 (14.3)	132.1 (15.9)	0.831	135.1 (12.4)	135.4 (13.2)	0.870	0.219
History of Hypertension = yes (%)	8 (32)	11 (44)	0.560	25 (33)	34 (45)	0.181	1
BP lowering Medications = yes (%)	7 (28)	9 (36)	0.762	24 (32)	32 (42)	0.264	0.631
History of Diabetes = yes (%)	0 (0)	0 (0)	NA	0 (0)	0 (0)	NA	1
Total cholesterol/HDL (mean (SD))	4.4 (1.0)	4.7 (1.1)	0.342	4.6 (1.2)	4.6 (1.2)	0.966	0.700
History of High Cholesterol = yes (%)	5 (20)	7 (28)	0.741	20 (27)	29 (39)	0.164	0.329
eGFR [mL/min/1.73m^2^] (mean (SD))	72.2 (10.3)	72.9 (14.0)	0.851	69.8 (9.4)	72.5 (11.9)	0.140	0.472
BNP [ng/L] (median [IQR])	22.1 [13.6, 30.4]	23.6 [16.3, 31.2]	0.808	21.5 [10.9, 29.3]	19.6 [12.6, 31.2]	0.656	0.145
NT-proBNP [ng/L] (median [IQR])	146.3 [100.6, 292.8]	204.7 [93.9, 319.7]	0.438	176.1 [90.5, 258.5]	142.6 [96.8, 294.9]	0.523	0.463
hsTNI [ng/L] (median [IQR])	2.5 [1.8, 5.2]	2.7 [1.9, 6.3]	0.554	2.8 [1.8, 4.1]	3.4 [2.4, 7.6]	0.015	0.513
CVD event type (%)			NA			NA	0.479
Death due to MI or CHD	0 (0)	3 (12)		0 (0)	3 (4)		
STEMI	0 (0)	2 (8)		0 (0)	10 (13)		
NSTEMI	0 (0)	7 (28)		0 (0)	24 (32)		
Unstable Angina	0 (0)	2 (8)		0 (0)	3 (4)		
Other Angina	0 (0)	2 (8)		0 (0)	13 (17)		
Ischemic Stroke	0 (0)	5 (20)		0 (0)	17 (22)		
Transient Ischemic Attack	0 (0)	4 (16)		0 (0)	6 (8)		
Days to first event (median [IQR])	NA	411.0 [244.0, 748.0]	NA	NA	1178.0 [791.2, 1432.5]	NA	< 0.001
Framingham Risk Score 10-year risk (median [IQR])	21.8 [15.9, 26.9]	24.2 [15.0, 30.3]	0.646	24.6 [15.8, 34.0]	25.4 [18.7, 31.9]	0.810	0.271
PREDICT-1° Risk Score 5-year risk (median [IQR])	10.2 [7.2, 12.0]	10.8 [8.2, 14.4]	0.455	10.3 [6.7, 14.2]	10.6 [7.0, 14.2]	0.437	0.747

*SD* standard deviation, *BMI* body mass index, *SBP* systolic blood pressure, *BP* blood pressure, *HDL* high density lipoprotein, *eGFR* estimated glomerular filtration rate, *BNP* B-type natriuretic peptide, *NT-proBNP* amino-terminal pro-hormone B-type natriuretic peptide, *hsTNI* high-sensitivity Troponin I, *ACS* acute coronary syndrome, *PREDICT-1*° NZ Primary Prevention Equations, *CoD* cause of death, *MI* myocardial infarction, *STEMI* ST-Elevation Myocardial Infarction, *NSTEMI* Non-ST-elevation myocardial infarction, *CHD* coronary heart disease. Where missing, values for the total cholesterol/HDL ratio (1 missing in Discovery cohort and 12 missing in Extended cohort) were set to the median value of the entire Hvols cohort (n = 3358), total cholesterol/HDL (women) = 3.82, total cholesterol/HDL (men) = 4.46. The NZ Deprivation quintile information was not available for this cohort and set to 3 for both men and women to calculate the PREDICT-1° Risk Score. Table generated using the ‘tableone’ (https://github.com/kaz-yos/tableone/) package within R/RStudio^17,18,39^.

### Immunoassays

The concentrations of three leading candidate plasma proteins were measured using commercial ELISA kits, including fetuin A (DFTA00 Human Fetuin A Quantikine ELISA Kit, R&D Systems, Minneapolis, MN, USA), hemoglobin (AB157707 Human Hemoglobin ELISA Kit, Abcam, Cambridge, MA, USA) and fibrinogen (AB208036 Human Fibrinogen SimpleStep ELISA Kit, Abcam), with each assay performed according to manufacturer’s instructions. All samples were run in duplicate, using a randomized, blocked experiment design to minimize bias due to inter-plate variability.

Protein concentration was calculated from a calibration curve using StatLIA (Brendan Technologies, Inc, Carlsbad, CA, USA)^19^. Samples were re-analyzed if the coefficient of variation between duplicates was ≥ 20%.

### Ethics approval and consent to participate

The study conformed to the Declaration of Helsinki and was approved by the New Zealand Health and Disability Ethics Committee (Reference CTY/01/05/062). All participants gave written, informed consent.

## Results

A flow chart describing participant selection criteria is shown in Fig. 1. The baseline characteristics of participants in the Discovery and Extended arms of the study are summarized in Table 1. There were no significant differences in baseline characteristics or cardiovascular risk factors between cases and controls in either the Discovery or the Extended sub-study, with the exception of hsTNI, which was higher in cases compared with controls in the Extended cohort (*p* = 0.015).

### Plasma mass spectrometric analysis

**Figure 2:**
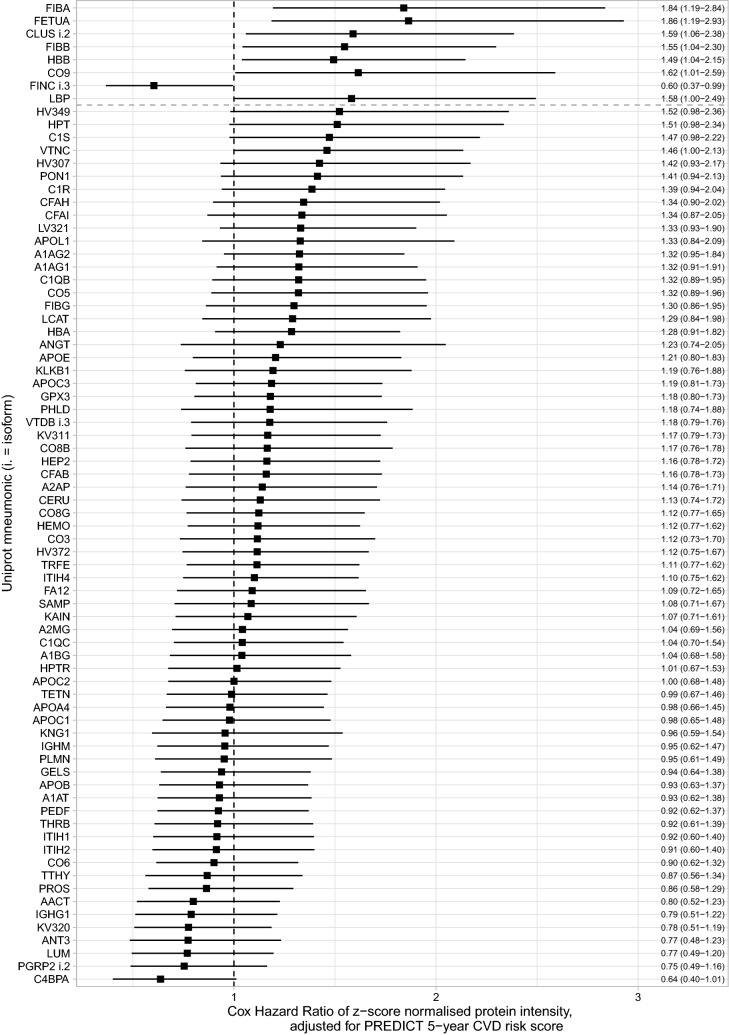
Cox Hazard Ratios of z-score standardized protein abundance in the Discovery cohort, adjusted for the log_2_-transformed PREDICT-1° 5-year CVD risk score. The whiskers represent 95% confidence intervals. The dashed vertical line corresponds to a Hazard Ratio of 1 (i.e. no difference of CVD risk based on this protein). The dashed horizontal line divides those proteins with *p* < 0.05 from those proteins with *p* 0.05. Z-score scaling of protein biomarker data was used instead of using raw protein mass spectrometric intensity to allow easy comparison between proteins. A one unit increase in z-score scaled data represents a one standard deviation increase in protein concentration. Protein IDs are the mnemonic of the uniprot ID (Supplementary Table 5). Figure generated using the ‘survival’ (https://github.com/therneau/survival) and ‘ggplot2′ (https://github.com/tidyverse/ggplot2) packages within R/RStudio^17,18,37,38^.

In the Discovery cohort, the 5-year risk PREDICT-1° score did not correspond to time-to-CVD-event (Cox model HR = 1.11 [0.79–1.56], p = 0.53), reflecting that the score alone was not able to distinguish those with subsequent CVD events from those who remained event free in this low-moderate risk sample. In the Discovery cohort, 76 proteins were robustly quantified in human plasma by DIA mass spectrometry. Of these, eight proteins/subunits were associated with CVD events independent of the PREDICT-1° score (Supplementary Table 2). The hazard ratios of these associations were (HR = hazard ratio [95% confidence interval] above vs below median): fibrinogen alpha chain (HR = 1.84 [1.19–2.84]); alpha-2-HS-glycoprotein (fetuin A) (HR = 1.86 [1.19–2.93]); clusterin isoform 2 (HR = 1.59 [1.06–2.38]); fibrinogen beta chain (HR = 1.55 [1.04–2.30]); hemoglobin subunit beta (HR = 1.49 [1.04–2.15]); complement component C9 (HR = 1.62 [1.01–2.59]), fibronectin isoform 3 (HR = 0.60 [0.37–0.99]); and lipopolysaccharide-binding protein (HR = 1.58 [1.00–2.49]), (all *p* unadjusted < 0.05, Fig. 2). On average, protein levels differed 1.2-fold (range 1.1- to 1.3-fold) between cases who had an event compared with controls who remained event free (Supplementary Table 2). Fibrinogen alpha chain, fetuin A, clusterin isoform 2 and hemoglobin were also independent of BNP, NT-proBNP, and hsTNI in Cox models (Supplementary Tables 3 & 4). Fetuin A and clusterin isoform 2 were highly correlated to one another (Supplementary Fig. 1) leading to the final choice of candidates, fibrinogen, fetuin A and hemoglobin, for further analysis via ELISA assay.

### ELISA versus DIA-MS assessment

**Figure 3:**
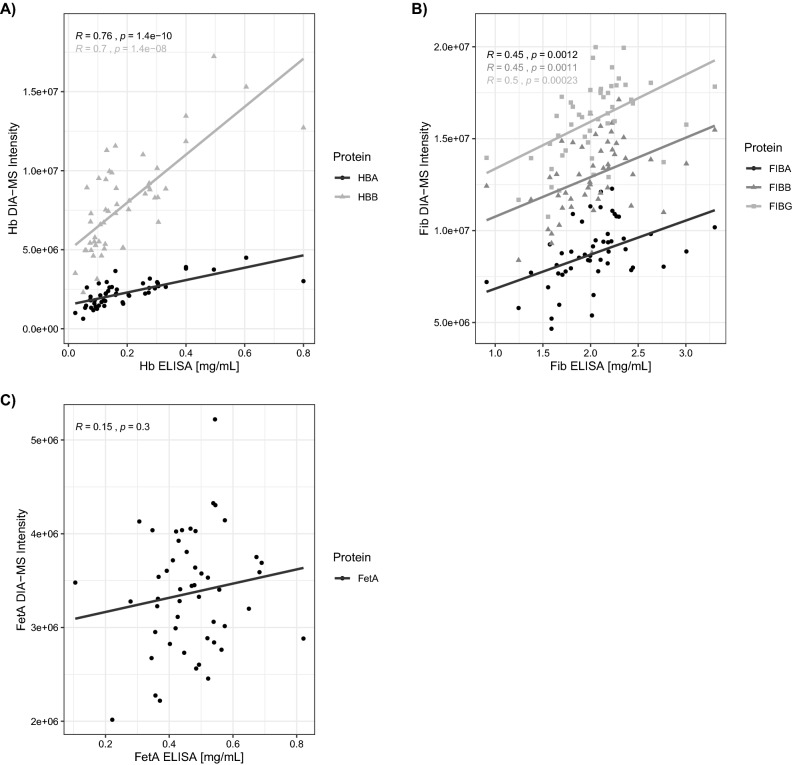
Scatterplots comparing protein concentrations measured via ELISA versus DIA-MS. Panel A1) hemoglobin (Hb) vs Hb alpha chain (HBA) and Hb beta chain (HBB). Panel B) fibrinogen (FIB) vs FIB chains alpha, beta, and gamma (FIBA, FIBB, FIBG) and Panel C) fetuin A (FetA). Correlations were assessed via Pearson for fibrinogen and fetuin A and via Spearman test for hemoglobin due to slight non-normal distribution of the HB ELISA data. Trendlines represent fits using linear regression models. Residual plots for model assessment provided as Supplementary Fig. 2. Figure generated using the ‘ggplot2′ (https://github.com/tidyverse/ggplot2) package within R/RStudio^17,18,37^.

The concentration of fibrinogen, hemoglobin and fetuin A in the same 50 samples from the Discovery arm were also measured using commercially-available ELISAs (Fig. 3). Limits of detection for DIA-MS were applied on each of the detected 9576 ions using IQR guided lower-end cut-offs (see Supplementary Methods). Our stringent quality control also included CV filtering of ions resulting in 1288 high-confidence ions for relative quantification with > 97% of these ions being detectable in each of the 50 people of the Discovery cohort. The coefficient of variation between duplicates measured by ELISA was ≤ 20% with all samples diluted appropriately to lie within the range of the standard curves. DIA mass spectrometry and ELISA methods were strongly correlated for hemoglobin (Spearman correlation *r* = 0.76 and 0.70, *p* < 0.05 for the hemoglobin alpha chain and the hemoglobin beta chain, respectively), and moderately correlated for fibrinogen (Pearson correlation *r* = 0.45, *r* = 0.45, *r* = 0.50, *p* < 0.05 for fibrinogen chains alpha, beta and gamma). However, no correlation was observed between DIA-MS and ELISA quantitation for fetuin A (Pearson correlation *r* = 0.15, *p* = 0.30). Therefore, we took both hemoglobin and fibrinogen (but not fetuin A) as candidates to validate our findings in the larger Extended cohort using ELISA assays.

### Extended cohort

There were statistically significant differences between the risk factor profiles of the Extended and the Discovery cohorts, including a longer duration between recruitment and first CVD event (median [IQR] = 1178.0 [791.2, 1432.5] days in the Extended cohort versus 411.0 [244.0, 748.0] days in the Discovery cohort, p < 0.001) and a higher body mass index (BMI) (26.4 ± 3.4 kg/m^2^ in Extended cohort versus 25.1 ± 2.7 kg/m^2^ in the Discovery cohort, p = 0.01) (Table 1). As was the case in the Discovery cohort, the PREDICT-1° score alone did not contribute significantly to risk prediction in our Cox models in the Extended cohort (HR = 1.16 [0.86–1.57], p = 0.33). In the Extended cohort, concentrations measured by ELISA of neither hemoglobin nor fibrinogen were significantly different between cases and controls. The measured hemoglobin concentration (median [IQR]) in controls was 141.5 µg/mL [103.3, 317.3] compared with 148.3 µg/mL [78.3, 304.0] in cases, *p* = 0.537. The fibrinogen concentration was 2.2 mg/mL [1.9, 2.6] in controls compared with 2.1 mg/mL [1.8, 2.4] in cases *p* = 0.099. When included in Cox-proportional hazard models, adjusted for PREDICT-1° 5-year CVD risk, neither fibrinogen nor hemoglobin showed significant associations with the time-to-event in Cox models (fibrinogen (HR = 0.86 [0.68–1.10]), p = 0.37 and hemoglobin (HR = 0.92 [0.73–1.15]), p = 0.59).

## Discussion

### Biomarkers to improve prediction

This study provides the first proof of principle that a DIA-MS proteomic approach can be used to identify additional circulating plasma biomarkers to improve current population-based screening approaches to predict future CVD events in asymptomatic individuals. The absence of predictive value of the PREDICT-1° score on its own in this low-to-moderate risk population sample demonstrates the need for improved models to estimate CVD risk. The three top ranked candidate plasma biomarkers from our DIA-MS Discovery screen (hemoglobin, fibrinogen and fetuin A) were independently associated with near-future CVD events (≤ 3 years) in asymptomatic individuals, independent of the PREDICT-1° score. However, in the Extended cohort, where the median time-to-event was considerably longer and individuals with a broader range of risk factors were included, neither hemoglobin nor fibrinogen were independently predictive of future CVD events in Cox-modelling. It is conceivable that these two proteins are associated with more immediate risk as the time to first event was ~ threefold shorter in the Discovery cohort (median of 244 days) compared with 791 days in the Extended Cohort, despite the Discovery cohort being characterised by a generally lower burden of other known risk variables than the Extended cohort (Table 1). Further, it is possible that the moderate correlation between detection techniques, DIA-MS and ELISA, contributes to the fact that the relationship between protein and CVD outcome observed in the Discovery cohort was not demonstrated in the Extended cohort. In general, a high correlation between ELISA and mass spectrometric results can be expected if the peptides used for quantification in DIA-MS and the detection of epitopes by ELISA antibodies allow equivalent detection of the analyte by the respective technology. However, there are many examples of mass spectrometry and ELISA not correlating strongly, oftentimes due to post-translational modification or due to cross-reactivity issues in ELISA^20–22^. Our findings indicate that certain circulating markers may improve risk prediction of impending CVD events in low-to-moderate CVD risk individuals.

Risk prediction models such as the NZ PREDICT-1° score, and the UK QRISK3 work best on the population level but suffer from poor discrimination at the level of the individual. Up to 20% of patients diagnosed with CVD have no traditional risk factors, and up to 40% have only one, suggesting that the accuracy of risk prediction models for individuals is modest and that CVD commonly occurs in people predicted to be at low-to-moderate risk^6^. Biomarkers may add value for individual risk stratification, especially for those in the low-to-moderate risk “grey zone,” where clinicians often do not have appropriate tools available to guide their decision making, and in those of older age (i.e. > 75 years). In our study of older individuals with a relatively low burden of cardiovascular risk factors, the NZ PREDICT-1° risk score was unable to discriminate between those who subsequently incurred a CVD event and those that remained event-free. Our findings highlight the feasibility of an unbiased DIA-MS approach for discovery of additional circulating plasma biomarkers that may add value to established risk factors and current approaches.

In the Discovery cohort we identified eight proteins (i.e. fibrinogen alpha chain, fetuin A, clusterin isoform 2, fibrinogen beta chain, hemoglobin subunit beta, fibronectin isoform 3, complement component C9, lipopolysaccharide-binding protein) that have potential to improve risk prediction of a near-future CVD event (within 3 years). Of these, fibrinogen is probably the best characterized in terms of its relation to CVD risk prediction. It has been previously proposed that circulating fibrinogen concentration be included in CVD risk stratification^23,24^. A previous study reported the age- and sex-adjusted hazard ratio per 1 g/L increase in usual fibrinogen concentration was 2.42 (95% CI, 2.24–2.60) for coronary heart disease; 2.06 (95% CI, 1.83–2.33) for stroke; 2.76 (95% CI, 2.28–3.35) for other vascular mortality and 2.03 (95% CI, 1.90–2.18) for nonvascular mortality^25^. Our data extend these findings by suggesting that fibrinogen remains independently predictive of outcomes even when adjusted for multiple other cardiovascular risk factors.

Hemoglobin circulating freely in the plasma and not contained within the erythrocyte has been implicated in pathological conditions, and has been proposed to contribute to abnormal production of highly reductive and oxidative compounds via the four heme ligands incorporated into each hemoglobin protein^26^. While not specifically associated with CVD risk, increased concentrations of cell-free hemoglobin have been linked to micro ruptures in the fragile vasculature of unstable atherosclerotic plaques^27^. In this context, hemoglobin has been reported to function as a “locally active disease modifier” and “intrinsic alarm molecule” (i.e. a biomarker) indicating bleeding and tissue destruction^28^.

The multifaceted biological role of Fetuin A has been recently reviewed and has been variably reported to be inversely correlated with coronary artery disease and atherosclerotic burden (presumed due to protection against vascular calcification) and positively correlated with coronary artery disease, possibly via its role in diabetes mellitus through the inhibition of insulin receptor tyrosine kinase^29^.

### Proteomics and biomarker discovery approaches

We have used a robust and unbiased analysis protocol for measuring plasma proteins in human plasma. Applying DIA mass spectrometry to biomarker discovery allows the identification and quantifation of > 300 proteins^30^. All of these proteins tend to be in the high- to medium-abundant concentration range in plasma, and so are readily detectable by a number of clinical assay platforms. However, depletion or further fractionation steps may be required to detect low-abundance proteins using DIA^31^. Plasma is the ideal, accessible sample reservoir for discovery of potential disease biomarkers, as it captures a considerable proportion of the proteome (~ 2,000 out of ~ 20,000 known proteins detectable using mass spectrometry, not considering isoforms)^32^. Furthermore, DIA approaches interrogating phosphorylation and glycosylation patterns, important for cell-signalling and protein function, are also being elucidated and likely to contribute further in the identification of novel biomarkers^33,34^. Continuous improvements in mass spectrometry technology, analysis pipelines and sample throughput are likely to contribute to future precision medicine biomarker discovery^35^.

The selectivity/specificity of mass spectrometry is superior to that of many ELISAs and also facilitates interrogation of post-translational modifications, which carry additional biological information that may be related to pathophysiology^36^. However, sensitivity of MS methods is still lacking compared with ELISA, especially for complex mixtures such as plasma where a small fraction of proteins dominates the contribution to the overall protein concentration. Additionally, ELISA is easily scalable with high-throughput easily implemented. DIA-MS methods are especially useful for tissue and cell-line proteomics, where the dynamic range of protein concentrations is smaller compared to that of plasma.

### Limitations

Our study has several limitations. First, by design we selected individuals with a relatively low burden of cardiovascular risk considering their older age, which limited the sample size of our Discovery and Extended cohorts. Second, our Discovery and Extended cohorts were not well-matched for time to first event (3 years vs. 5 years) and cardiovascular risk, reducing our ability to replicate our findings. Third, the heterogeneity of outcomes and the different proportions of each outcome in the Discovery and the Extended cohorts may reduce power to detect biomarker associations with specific outcomes. Fourth, despite the good correlation in the Discovery phase, the two techniques (DIA-MS and ELISA) have inherent differences affecting their specificity in terms of which part of the protein is detected. Fifth, the presence of abundant plasma proteins in our samples may have prevented detection of less abundant proteins with similar or stronger associations with CVD events. The MS-detectable plasma proteome is approximately 2000 proteins^32^, of which 76 were robustly quantified across the 50 DIA samples in this study. This is approximately 28% of the number of proteins observed in other studies (272 robustly quantified proteins) using MS1-level BoxCar quantification. This reflects differences in mass spectrometric data acquisition methods and quality filtering for inclusion/exclusion of peptides into the final data-set^32^.

## Conclusions

DIA-MS methods have great potential in biomarker discovery/hypothesis generation due to measuring many proteins simultaneously. Using DIA-MS we have identified eight protein biomarkers that may be independently associated with near-future CVD events (< 3 years) in asymptomatic individuals of older age with a relatively low cardiovascular risk.

## Supplementary information

Supplementary information 1.

Supplementary information 2.

## Data Availability

The datasets generated during and/or analysed during the current study are available from the corresponding author on reasonable request.

## Abbreviations

DIA-MS: Data independent acquisition mass spectrometry
HR: Hazard ratio
CVD: Cardiovascular disease
IHD: Ischemic heart disease
FRS: Framingham risk score
NZ: New Zealand
ECG: Electrocardiograph
PREDICT-1°: New Zealand primary prevention equations
QRISK3: United Kingdom prediction algorithm for cardiovascular disease
UK: United Kingdom
TC: Total cholesterol
HDL: High-density lipoprotein
LDL: Low-density lipoprotein
HbA1C: Glycated hemoglobin
BNP: B-type natriuretic peptide
NT-proBNP: Amino-terminal pro-hormone B-type natriuretic peptide
TNT: Cardiac troponin T
TNI: Cardiac troponin I
HF: Heart failure
MI: Myocardial infarction
ELISA: Enzyme-linked immunosorbent assay
Hvols: Canterbury Healthy Volunteers cohort
hsTnI: High-sensitivity Troponin I
SWATH-MS: Sequential windowed acquisition of all theoretical fragment ion spectra mass spectrometry
IQR: Interquartile range
BMI: Body mass index
SD: Standard deviation
SBP: Systolic blood pressure
BP: Blood pressure
eGFR: Estimated glomerular filtration rate
ACS: Acute coronary syndrome
CoD: Cause of death
STEMI: ST-elevation myocardial infarction
NSTEMI: Non-ST-elevation myocardial infarction
CHD: Coronary heart disease
Hb: Hemoglobin
HBA: Hemoglobin alpha chain
HBB: Hemoglobin beta chain
FIB: Fibrinogen
FIBA: Fibrinogen alpha chain
FIBB: Fibrinogen beta chain
FIBG: Fibrinogen gamma chain
