# Supplementary material for: Fibrinogen and hemoglobin predict near future cardiovascular events in asymptomatic individuals
Source: Sci Rep. 2021 Feb 25;11:4605. doi: 10.1038/s41598-021-84046-7 (PMC7907085; doi:10.1038/s41598-021-84046-7)
Supplement: Supplementary file 1 — Supplementary information 1. [file 41598_2021_84046_MOESM1_ESM.docx]

Fibrinogen and hemoglobin predict near future cardiovascular events in asymptomatic individuals

**M Lassé^1^, AP Pilbrow^1^,** **T Kleffmann^2^, EA Överström^1^, A von Zychlinski^3^, CM Frampton^1^, KK Poppe^4^, RW Troughton^1^, LK Lewis^1^, TCR Prickett^1^, CJ Pemberton^1^, AM Richards^1,5^, VA Cameron^1^.**

^1^Christchurch Heart Institute, Department of Medicine, University of Otago, Christchurch, New Zealand;

^2^Department of Biochemistry, University of Otago, Dunedin, New Zealand;

^3^Southern Community Laboratories Ltd, Dunedin, New Zealand

^4^School of Population Health, Faculty of Medical and Health Sciences, University of

Auckland, Auckland, New Zealand

^5^Cardiovascular Research Institute, National University of Singapore, Singapore

# Supplementary Information

## Supplementary Figures


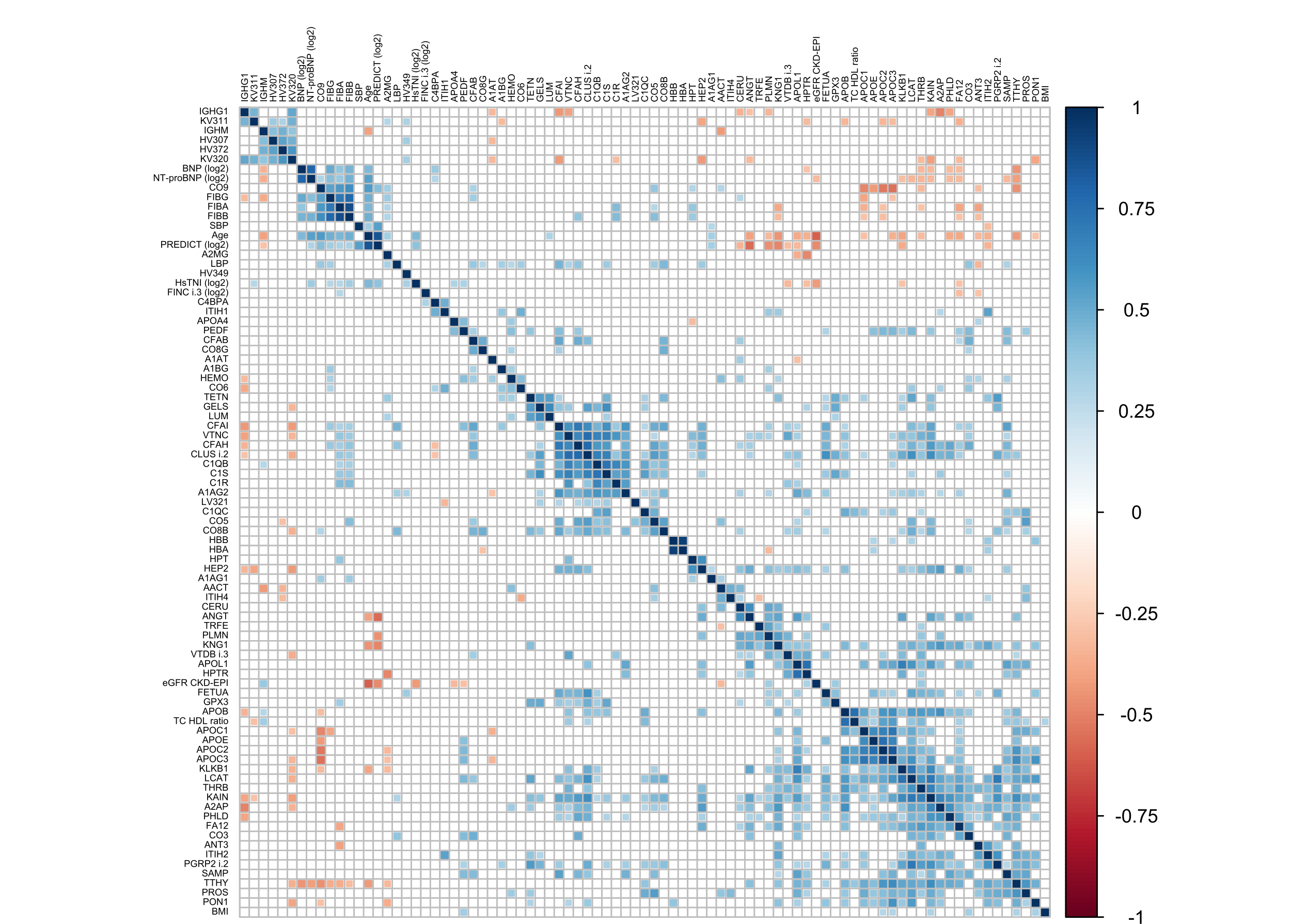


Supplementary Figure 1

Correlation matrix of 77 plasma proteins including hemoglobin (HBB and HBA), fibrinogen (FIBA, FIBB, and FIBG), and fetuin A, together with clinical risk factors relevant to cardiovascular disease and the PREDICT-1° 5-year CVD risk score. Blue indicates a positive correlation, red indicates a negative correlation. Blank (no colour means no statistically different correlation, p≥0.05). Hierarchical clustering method “Ward” was used. Any data displaying a skewed distribution were log_2_-transformed prior to analysis. Figure generated using the ‘corrplot’ (https://github.com/taiyun/corrplot) package within R/RStudio (1-3).


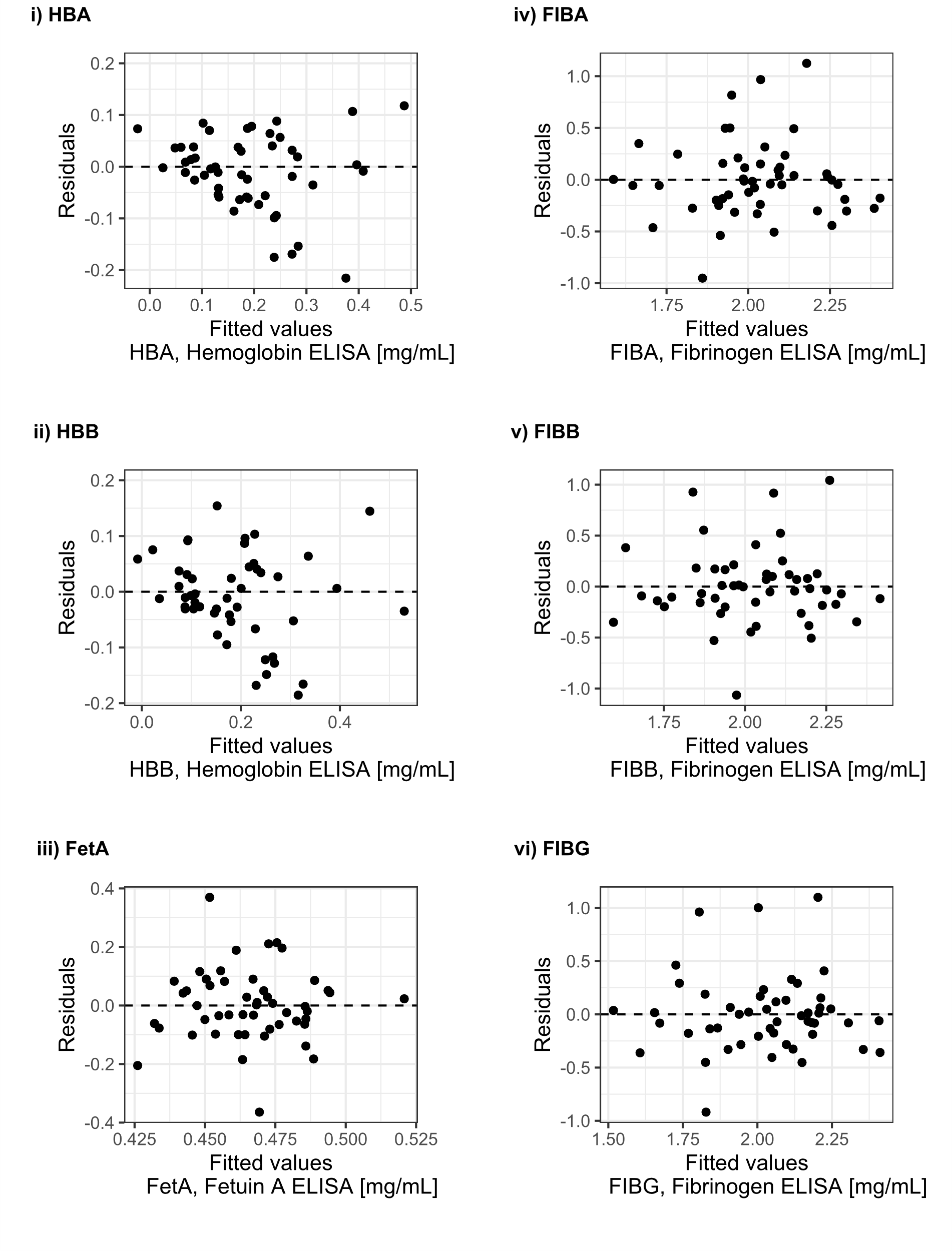


Supplementary Figure 2

Residual plots assessing the linear model of ELISA and DIA-MS. Applying the linear regression model is appropriate here as indicated by random scatter around the horizontal dashed line. Dashed lines represent perfect fit, points above the line represent under-estimation by the model and points below the line represents an overestimation of the model. Figure generated using the ‘ggplot2’ package (https://github.com/tidyverse/ggplot2) within R/RStudio (1, 3, 4).

## Supplementary Methods

### Detailed Sample Preparation

Plasma (2 µL) was denatured stepwise using 60 µL of denaturing solution 1 containing sodium deoxycholate (DOC) followed by 82 µL of denaturing solution 2 containing acetonitrile, calcium chloride, and ammonium bicarbonate (ABC) (Supplementary Table 1). Samples were incubated for 5 minutes and briefly vortexed between each step. Final concentrations of solutes were 15% ACN, 0.5% DOC, 1 mM CaCl_2_ and 100 mM ABC. Denatured samples were hydrolysed with trypsin. After incubation for 3 hours at 42°C, more trypsin was added (i.e. trypsin boost) and the samples were incubated at 37°C overnight. The protein to trypsin ratio was ~1:30 (w/w), with an estimated plasma protein concentration of 60-70 mg/mL. Digestion was stopped and DOC precipitated simultaneously by addition of formic acid to a final concentration of 0.2%. Tryptic peptides were purified with Vydac C18 Silica 96-well MACROSpin plates (SNS SS18V-L, The Nest Group Inc.). Retention time calibration peptides (iRT-Kit, Biognosys) were spiked into each sample at 75x dilution of the iRT stock solution (final sample volume was 30 µL) to correct for relative retention time differences between runs (Escher et al, 2012). Reduction and alkylation of cysteine residues were omitted as prior tests showed a higher number and more consistent protein identification, albeit at the cost of lower average sequence coverage per protein and lower average number of validated peptide spectra (data not shown).

### Detailed DIA library generation

A comprehensive spectral library was generated from a pooled plasma sample made up of 22 µL of plasma from each of the 50 participants in the Discovery arm. The pooled sample (1 mL) was depleted of highly-abundant proteins with the ProteoMiner kit according to manufacturer’s instructions. The depleted protein pool was buffer exchanged first into 8 M urea in 100 mM ABC before buffer exchange into 100 mM ABC using Amicon Ultra-0.5 Centrifugal Filter Devices (10 kDa molecular weight cut-off) and trypsinized (volumes were adjusted to account for 1 mL of depleted plasma (protein depletion factor ~50x)). The pooled, depleted and trypsinized library sample was fractionated using off-gel isoelectric focusing (Agilent OFFGEL Fractionator, Agilent Technologies, Santa Clara, CA, USA) into 12 fractions along a gradient from pH4 to pH10 following manufacturer’s instructions. Fractions were purified using C18 and analysed in technical injection duplicates by nanoflow liquid chromatography-coupled tandem mass spectrometry.

Spectral library shotgun data of depleted and fractionated peptides were acquired on an AB Sciex 5600+ TripleTOF mass spectrometer coupled to an ekspert^TM^ nanoLC 415 system (eksigent, AB Sciex, Dublin, CA, USA). Each IEF fraction (6 µl) was injected onto a 20 cm reverse-phase emitter tip column (75 µm ID, packed in-house with 3µm C-18 beads) and separated using a 2-hour acetonitrile gradient of mobile phase B (acetonitrile (90% v/v) and formic acid (0.1% v/v) in water) in mobile phase A (acetonitrile (2% v/v) and formic acid (0.1% v/v) in water) at a flow rate of 400 nL/minute, using the following gradient steps: i) 3 min at 5%, ii) 90 min from 5% to 25%, iii) 10 min from 25% to 40%, iv) 10 min from 40% to 95%, v) 1 min at 95%, vi) 1 min from 95% to 5% and vii) 5 min at 5%.

Mass spectrometric data were acquired by data dependent acquisition (DDA) in positive ion mode using tandem mass spectrometry (MS1/MS2). Peptide precursor survey scan spectra were accumulated using the first mass analyzer (MS1) in the m/z range of 400-1,300 with an ion accumulation time of 200 milliseconds (ms). Within each MS1 cycle, the 20 most intense precursor ions with charge state +2 to +5 were selected for fragmentation and second mass analysis (MS2) in high sensitivity mode to capture peptide fragments. MS2 spectra were collected in the m/z range of 100-3000 with 150 ms accumulation time for each of the 20 precursors resulting in a maximum cycle time of 3.25 seconds. Each precursor ion from MS1 was allowed to be selected twice for MS2 and then dynamically excluded from reselection for 90 seconds.

DDA data (*.wiff format) were directly read into ProteinPilot (V5.0.1, AB Sciex) and MS2 spectra were searched against the reviewed UniProt database *(5)* for human proteins (including isoforms, January 2017) appended with reversed sequence decoys *(6)* (84,294 protein and isoform sequences including decoys). The Paragon^TM^ search algorithm criteria for peptide identification were: Sample Type: Identification, Cys alkylation = None, Digestion = Trypsin, Instrument = TripleTof 5600, Special Factors = None, Species = None, ID Focus = including Biological modifications, Search Effort = Thorough ID. The Protein detection threshold was set to 10% to enable estimation of false discovery rate (FDR) on protein, peptide, and ion level respectively *(7)*, based on a target-decoy scoring system. The final library consisted of 361 proteins with an FDR <1%, which were selected for further processing with the PeakView MicroSWATH app. The retention time for each peptide was calibrated using a mix of retention time calibrator peptide standards (iRT Kit, Biognosys AG (Schlieren, Switzerland) and ApoA1 peptides (see Supplementary Table 7).

The Paragon^TM^ search of mass spectrometric acquisition of shotgun runs of the IEF fractions of the pooled depleted plasma sample yielded 76,465 spectra. This yielded 16,361 distinct peptides and 361 detected proteins at 1% FDR, which were used as the spectral library for all subsequent DIA-MS analyses. The number of detected proteins for non-depleted plasma samples is usually 200 - 300 *(8)*.

### DIA-MS of Discovery Cohort

DIA-MS of the 50 individual (unfractionated), trypsinized plasma samples was undertaken on the same LC-coupled TripleTof 5600+ instrument using the same 2-hour gradient as the shotgun analyses. DIA-MS spectra were acquired using variable window width for precursor ion selection. Window sizes were calculated using the AB Sciex SWATH Variable Window Calculator (V1.0) based on the MS1 signal spread of prior DDA runs. A total of one MS survey scan (50 ms accumulation time) and 34 SWATH-MS2 scans with overlapping m/z windows (1 m/z for the window overlap) were used, covering the precursor mass range of 400-1,250 m/z (Supplementary Table 6). SWATH-MS2 spectra were collected from 100-2,000 m/z. The collision energy spread (CE) was optimized automatically for each window using a CE spread of 15 eV. An accumulation time (dwell time) of 96 ms was used for all fragment ion scans in high-sensitivity mode, resulting in a duty cycle of ~3.4 seconds. For every sample, three repeated injections and SWATH-MS measurements were performed (50 biological replicates, each with 3 technical injection replicates and no sample preparation replicates). To mitigate batch effects, cases or controls were randomized separately, and then the injection sequence alternated between cases and controls.

### Analysis of DIA-MS data

Analysis was carried out with AB Sciex software (SWATH Acquisition™ MicroApp in PeakView® Software) and R. The *.group output file of the DDA search in ProteinPilot was loaded as the ion library excluding shared peptides (non-proteotypic peptides) *(9)*. Retention times for all 150 DIA-MS *.wiff files (50 biological samples with 3 technical injection replicates each) were aligned using a combination of retention time standard peptides from Biognosys and ApoA1 (Supplementary Table 7).

DIA-MS Processing settings in the SWATH Acquisition™ MicroApp were set to include 6 peptides per protein, 6 transitions per peptide, FDR Threshold of 1%, fixed library rank of peptides, an 18 minutes window for the extracted ion Chromatogram (XIC) and a mass accuracy of 75 ppm *(10)*. Similar to DDA analysis, each peptide is assigned an FDR based on a target-decoy scoring system, with both target and decoy sequences scored on chromatographic and spectral features.

Ion level data were analysed using R and RStudio *(1)* in the following sequence. Each of the 150 runs (50 patient samples in technical injection replicates) were normalized/scaled using area under the curve normalization. A limit of detection was set for each ion, with any low-end outliers being excluded if ion intensity was smaller than Q1 - (1.5 * IQR) among the 150 runs. The coefficient of variation (CV) of the technical triplicates was calculated for each ion and a cut-off value applied (ion CV < 25 in at least 80% of patient samples (40 out of 50 samples)). For any individual sample, ions were only kept in the data set if the ion was observed in ≥2 of the three technical injection replicates. For each technical triplicate, the median intensity of each ion was extracted as the “final” ion intensity for the respective sample. The median intensity for each ion was used to sum ions to peptide areas and subsequently peptide to protein areas. This approach allows for reliably estimating global protein-level changes *(11-13)*. After summing to protein level, the distributions of protein data were assessed *via* histograms and QQ-plots. Non-normally distributed proteins (only fibronectin isoform 3) were log_2_-transformed and statistical analysis was carried out on protein level data. In total, 76 unique proteins were robustly quantified using our pipeline and used for subsequent analyses. Abundances and relative concentrations of proteins using our pipeline were comparable with DIA data analysis software mapDIA *(14)* (100 proteins quantified) and MSStats *(15)* (123 proteins quantified). Protein biomarker candidates were selected from amongst the top 20 ranked proteins in at least two of the three analysis pipelines (in-house, MSStats, mapDIA). Ranking was done by p-value (in-house analysis), q-value (MSstats), or score (mapDIA). Further inferential statistical tests were then conducted using protein-level data from the in-house analysis pipeline.

### Biomarker Candidate Shortlisting Pipeline

Protein candidates were selected if they were predictive of subsequent cardiovascular events independent of the (log_2_-transformed) PREDICT-1° 5-year CVD risk score *(16)* (p<0.05). Protein candidates were ranked on their hazard ratios (calculated from z-score scaled protein intensities to allow direct comparison of proteins). Correlation matrices were used to ensure that the final candidates were not strongly correlated with each other, to minimize multi-collinearity in any models.

### Data analysis of ELISAs

For each ELISA assay, protein concentration was derived from the mean absorbance of duplicate readings fitted to a 4-parameter logistic regression standard curve using StatLIA *(17)*. Statistical analysis was performed with R. To assess the validity of measuring the concentration of protein markers identified via DIA by ELISA we compared the two techniques via residual plots. Applying a linear regression model (i.e. correlation between ELISA and DIA) is appropriate for the two markers hemoglobin and fibrinogen as indicated by random scatter around the horizontal dashed line in the residual plots (supplementary Figure 2). To test for differences in baseline characteristics between cases and controls, Chi-Square testing was used for categorical variables, Welch one-way testing for normally distributed continuous variables and the Kruskal-Wallis rank-sum-test for non-normally distributed continuous variables using the R package ‘tableone’ *(18)*. Any protein concentration data displaying skewed distributions were log_2_-transformed prior to analysis. Protein concentrations were compared between cases and controls with ANOVA. To describe the linear relationship between protein concentration and continuous clinical parameters Pearson and Spearman correlation tests were carried out using the R package ‘psych’ *(19)*. Associations between protein concentration and time to cardiovascular event were assessed with Cox-proportional hazards analysis using the R package ‘survival’ *(20)*. A p-value <0.05 was taken to indicate statistical significance.

## References for Methods

1. R Core Team. R: A language and environment for statistical computing. 2018.

2. Wei T, Simko V, Levy M, Xie Y, Jin Y, Zemla J. Package ‘corrplot’. Statistician 2017;56:e24.

3. Team R. Rstudio: Integrated development environment for r (2016). Boston, MA 2020.

4. Wickham H. Ggplot2: Elegant graphics for data analysis. springer; 2016.

5. The UniProt Consortium. Uniprot: A worldwide hub of protein knowledge. Nucleic Acids Research 2018;47:D506-D15.

6. Elias JE, Gygi SP. Target-decoy search strategy for increased confidence in large-scale protein identifications by mass spectrometry. Nat Meth 2007;4:207-14.

7. Tang WH, Shilov IV, Seymour SL. Nonlinear fitting method for determining local false discovery rates from decoy database searches. Journal of Proteome Research 2008;7:3661-7.

8. Krisp C, Molloy MP. Swath mass spectrometry for proteomics of non-depleted plasma. In: Greening DW, Simpson RJ, editors. Serum/plasma proteomics: Methods and protocols New York, NY: Springer New York; 2017. p. 373-83.

9. Mallick P, Schirle M, Chen SS, Flory MR, Lee H, Martin D, et al. Computational prediction of proteotypic peptides for quantitative proteomics. Nature Biotechnology 2007;25:125-31.

10. Bjelosevic S, Pascovici D, Ping H, Karlaftis V, Zaw T, Song X, et al. Quantitative age-specific variability of plasma proteins in healthy neonates, children and adults. Molecular & Cellular Proteomics 2017;16:924-35.

11. Niu L, Geyer PE, Wewer Albrechtsen NJ, Gluud LL, Santos A, Doll S, et al. Plasma proteome profiling discovers novel proteins associated with non‐alcoholic fatty liver disease. Molecular systems biology 2019;15:e8793.

12. Liu Y, Buil A, Collins BC, Gillet LC, Blum LC, Cheng LY, et al. Quantitative variability of 342 plasma proteins in a human twin population. Molecular systems biology 2015;11:786.

13. Ludwig C, Claassen M, Schmidt A, Aebersold R. Estimation of absolute protein quantities of unlabeled samples by selected reaction monitoring mass spectrometry. Molecular and Cellular Proteomics 2012;11.

14. Teo G, Kim S, Tsou C-C, Collins B, Gingras A-C, Nesvizhskii AI, Choi H. Mapdia: Preprocessing and statistical analysis of quantitative proteomics data from data independent acquisition mass spectrometry. Journal of proteomics 2015;129:108-20.

15. Choi M, Chang C-Y, Clough T, Broudy D, Killeen T, MacLean B, Vitek O. Msstats: An r package for statistical analysis of quantitative mass spectrometry-based proteomic experiments. Bioinformatics 2014.

16. Pylypchuk R, Wells S, Kerr A, Poppe K, Riddell T, Harwood M, et al. Cardiovascular disease risk prediction equations in 400000 primary care patients in new zealand: A derivation and validation study. The Lancet 2018;391:1897-907.

17. Brendan Technologies I. Statlia enterprise. Vol. 3.2 Ed. Carlsbad, CA, USA, 2004.

18. Yoshida K, Chipman JJ, Bohn J, McGowan LD, Barrett M, Christensen RHB. Package ‘tableone’. 2019.

19. Revelle WR. Psych: Procedures for personality and psychological research. 2017.

20. Therneau TM, Lumley T. Package ‘survival’. R Top Doc 2015;128.
